# Introduction and Comparison of Novel Decentral Learning Schemes with Multiple Data Pools for Privacy-Preserving ECG Classification

**DOI:** 10.1007/s41666-023-00142-5

**Published:** 2023-08-17

**Authors:** Martin Baumgartner, Sai Pavan Kumar Veeranki, Dieter Hayn, Günter Schreier

**Affiliations:** 1 Center for Health & Bioresources, AIT Austrian Institute of Technology, Giefinggasse 4, 1210 Vienna, Austria; 2 Institute of Neural Engineering, Technical University of Graz, Graz, Austria; 3 Ludwig Boltzmann Institute for Digital Health and Prevention, Salzburg, Austria

**Keywords:** Decentral learning, Privacy-preserving artificial intelligence, Machine learning, Deep learning, Decision-support

## Abstract

Artificial intelligence and machine learning have led to prominent and spectacular innovations in various scenarios. Application in medicine, however, can be challenging due to privacy concerns and strict legal regulations. Methods that centralize knowledge instead of data could address this issue. In this work, 6 different decentralized machine learning algorithms are applied to 12-lead ECG classification and compared to conventional, centralized machine learning. The results show that state-of-the-art federated learning leads to reasonable losses of classification performance compared to a standard, central model (−0.054 AUROC) while providing a significantly higher level of privacy. A proposed weighted variant of federated learning (−0.049 AUROC) and an ensemble (−0.035 AUROC) outperformed the standard federated learning algorithm. Overall, considering multiple metrics, the novel batch-wise sequential learning scheme performed best (−0.036 AUROC to baseline). Although, the technical aspects of implementing them in a real-world application are to be carefully considered, the described algorithms constitute a way forward towards preserving-preserving AI in medicine.

## Introduction

### Artificial Intelligence in Healthcare

Artificial intelligence (AI), in particular, the fields of machine learning (ML) and its advancement deep learning, has led to prominent and spectacular innovations in various medical fields such as radiology pathology [1], genomics [2], injury risk assessment [3], and disease prognosis [4, 5]. AI applications are expected to play an increasing role in the future of everyday medicine, based on (a) superior processes as compared to the state-of-the-art with better outcomes for patients and (b) non-inferior processes which are less expensive in terms of costs, time, and/or resources.

Various techniques exist in the field of machine learning, which are currently dominated by artificial neural networks (ANNs). Most recently, the introduction of residual neural networks by He et al. in 2016 [6] has revolutionized this field. Residual models have shown astonishing results in various fields, often outperforming other architectures. At the Computing in Cardiology/PhysioNet Challenge in 2020 [7], 9 out of the 10 [8–17] best performing competing teams have used some type of residual network or skip connections. Regardless of the chosen technology or architecture, there is one aspect all AI algorithms have in common: the need for data. The correlation between data availability and model quality has been documented [18–20] and is now broadly accepted by the research community. Recently, there appears to be a shift in the literature to focus more on the data aspect of AI. In 2022, AI pioneer Andrew Ng shared this sentiment by stating that data should be the central element of AI applications, not the models per se [21].

### Artificial Intelligence and Clinical Data

Any data, especially health data, are subject to rigorous legal regulations. Additionally, medical data is often collected in decentralized settings. Institutions like hospitals or medical universities collect data from their patients for routine care applications and/or for clinical trials and other research activities. However, beyond the scope of this primary use, the data is rarely used for other purposes (secondary use) let alone shared with other institutions. Due to legal regulations, sharing of data with other institutions is often related to certain risks for the data holders and owners. However, if the highest level of privacy preservation was applied to all AI applications, the utility of the data would be reduced, and severe impairments of the clinical outcome would need to be conceded. This applies especially for applications that either require extensive amounts of data or areas where data is extremely sparse, such as rare diseases. Therefore, methods that balance data protection against data availability to optimize the overall outcome (“privacy-preserving AI”) are urgently needed.

### Privacy-Preserving Artificial Intelligence

Typically, clinical data is anonymized or pseudonymized prior model development. However, it has been shown in multiple studies that removing obvious identifying elements (e.g., names, date of birth, addresses) is not sufficient to protect the patients’ privacy, since these datasets are still highly vulnerable to re-identifying attacks [22–25]. Latanya Sweeney found that with the three basic quasi-identifiers of date of birth, zip code, and gender, 87% of individuals can be successfully re-identified [26]. Re-identification is possible by cross-referencing the remaining information with other, publicly available or leaked data, which is ever increasing. One could theoretically remove even more information from the datasets to address this, but at the same time, the utility of that data is decreased. In her speech at the Differential Privacy Symposium in 2016, Cynthia Dwork famously stated “De-identified data isn’t” [27], aptly summarizing this dilemma of utility versus privacy. The concepts of k-anonymity [28] and l-diversity [29] are allowing for a gradual removal of sensitive information to address this, but are still vulnerable to attacks (e.g., skewness or similarity attacks) [30].

Various alternative methods have been explored in recent publications to centralize the data while still sufficiently preserving privacy. However, discrepancies between promising academic ideas and practically applicable solutions exist. There are prominent examples to this issue:

•Homomorphic encryption is a compelling technique, which allows operations on fully encrypted data without prior decryption. Craig Gentry published the first fully homomorphic encryption scheme in 2009 [31]. While the technology is certainly promising, it is currently still computationally too expensive for widespread practical application in most clinical applications.

•Dwork et al. introduced differential privacy [32], which has been successfully implemented in a wide range of applications [33]. To be differentially private, a database is transformed so that the individual records are obscured, but the underlying statistical information is retained. However, this approach might not be applicable on small datasets [34, 35].

•Another approach that results in a similar solution is the application of generative adversarial networks (GANs) [36], which produce synthetic data samples derived from original examples. Those samples exhibit the same statistical properties while not containing real private information. This approach has already been applied on medical data [37, 38]. However, GANs are computationally expensive, time-consuming, and their output is notoriously difficult to validate.

Instead of trying to find secure methods to aggregate data, federated learning (FL) is aiming to centralize knowledge without ever collecting data in a central infrastructure. The concept was proposed by Google researchers McMahan et al. in 2015 for improving typing predictions in their Android operating system [39].

### Federated Learning–Principles

The core principle of FL is to avoid the pooling of data from different participating clients (distinct participants with their individual datasets are referred to as *nodes* in further writing) into a central point of infrastructure. In FL, data stays in the nodes’ secure local environment where they were collected, and knowledge exchange is realized by transferring models and models only. This eliminates the need of secure data transfer and storage, which always comes with a high risk of data leakages. In its original proposal [39], the FL workflow is comprised of five steps:

1. a central model is created
2. the model is distributed to all nodes
3. this model is trained at the clients’ infrastructure with the respective local data only
4. the changes in the models’ parameters are securely averaged
5. the central model is updated with the new parameters

Steps 2–5 are repeated multiple times until the model converges. Neural networks are well-suited for this as they are comprised of large matrices of weights and biases, for which standard mathematical operations like calculating a mean are easily applicable. McMahan et al. proposed averaging the parameters in a buffer, to obfuscate the individual nodes’ contribution even further [40].

A 2020 Nature publication investigated the possibility of applying federated learning in medicine and underlined its importance and potential [41]. However, the application of existing FL approaches in different scenarios might come with new challenges. In this paper, we focus on a simulated scenario, in which learning is not delegated to individual patients, but to various institutions holding pooled sets of data. In this scenario, only a few data nodes are used for training, while in its original application, potentially millions of Android smartphones were available. Furthermore, the different nodes can potentially provide rather homogenous datasets as not only the type of health data provider can vary (hospitals, research institutions, sports rehabilitation centers, geriatric homes, etc.) but also the population from which the data were collected (healthy subjects, patients, elderly adults, etc.). Another important aspect is dataset size since the quantity of data used in training is a well-established indicator of machine learning model performance. A yet unexplored question is whether the contribution of small institutions to a FL network could potentially have a negative effect on the final model’s performance. These considerations raise the question if averaging the models’ weights is truly sufficient in such a setting, as described by Rieke et al. [41]. Sheller et al. proposed three alternative methods of federated learning for application in medicine: Federated Learning, Institutional Incremental Learning and Cyclic Institutional Incremental Learning [42]. In their experiments, they compared a sequential learning scheme to the conventional federated learning approach and centralized machine learning. They found that cycling over institutions during learning achieved results comparable to conventional federated learning and even to centralized machine learning. However, this approach was less stable.

### Aims and Scope

The aim of the present work is to develop new decentral learning schemes for a scenario in which data is distributed across different sources. To simulate a realistic setting, four distinct open-source electrocardiogram (ECG) datasets of different size and with different characteristics were chosen to serve as nodes (as described in chapter 1.4) in the experiments. The learning schemes are applied to these datasets in a complex multi-label, multi-class classification task. Their performance is compared to a standard machine learning approach, in which data is centralized. The main questions to be answered in this study are as follows: (A) can standard federated learning as a form of privacy-preserving AI be applied to medical data with few nodes and (B) do methods exists that might improve the standard federated learning algorithm as described in the chapter above?

## Methods

### Data Description

We used the data provided for the 2020 Computing in Cardiology/PhysioNet challenge [7], which consisted of six publicly available 12-lead ECG datasets (CPSC, CPSC-Extra, INCART, PTB, PTB-XL, Georgia). Datasets collected by the same institutional source (CPSC and CPSC-Extra; PTB and PTB-XL) were merged to simulate a realistic distributed learning setting, resulting in a total number of four nodes: (1) INCART, (2) CPSC, (3) Georgia, and (4) PTB (Table 1).

**Table 1:** Data description of the four ECG datasets used for our analyses

Node ID	Name	Number of samples	Duration (s)	Sampling rate (Hz)	Mean age (y)	Female (%)	Number of classes	Reference
1	CPSC	10,330	6–144	500	61.36	46.35	73	[43]
2	Georgia	10,344	5–10	500	60.52	46.34	67	[7]
3	INCART	74	1800	257	55.99	45.95	37	[44]
4	PTB	22,353	10–120	500, 1000	59.76	47.41	60	[44, 45]

The ECG recordings in these databases were heterogeneous in terms of signal length, sampling rate, demographic properties, and the number of classes. To imitate the participation of a smaller institution with less data, the INCART set (patient base *n* = 74) was included, which is the most different from the other sets. Furthermore, the INCART ECGs were longer (30 min), and patients tended to be younger (mean age = 55.99 years). In total, 111 different classes represented by SNOMED codes were present. Each ECG could be labelled with one or multiple classes. For this publication, medically related classes were joined or merged into parent categories, resulting in 13 classes as described in Table 2.

**Table 2:** Considered ECG classes and frequency of occurrence in each data source

Class	CPSC	Georgia	INCART	PTB	Total
Sinus rhythm	922	1752	0	18,172	20,846
ST interval abnormal	2985	3053	10	2188	8236
Myocardial infarction	1544	7	9	5629	7189
T wave abnormal	27	3118	1	2639	5785
Myocardial ischemia	545	1635	0	2580	4760
Right bundle branch block	2057	977	2	1660	4696
Left ventricular hypertrophy	158	1232	10	2359	3759
Atrial fibrillation	1374	570	2	1529	3475
Bradycardia	316	1683	11	637	2647
Ventricular ectopics	896	398	49	1,154	2497
Tachycardia	303	1261	11	827	2402
1st degree AV block	828	769	0	797	2394
Atrial ectopics	742	640	7	555	1944

### Pre-processing

All recordings were resampled to 250 Hz. Subsequently, from each signal, a 10-s sequence was extracted to generate a uniform data sample for the machine learning model. The first 5 s of a signal were ignored in this selection if excessive data was available. The ECG data was filtered by a bandpass filter (3–30 Hz, Butterworth bandpass, 2nd order).

### Model Architecture Description

**Fig. 1:**
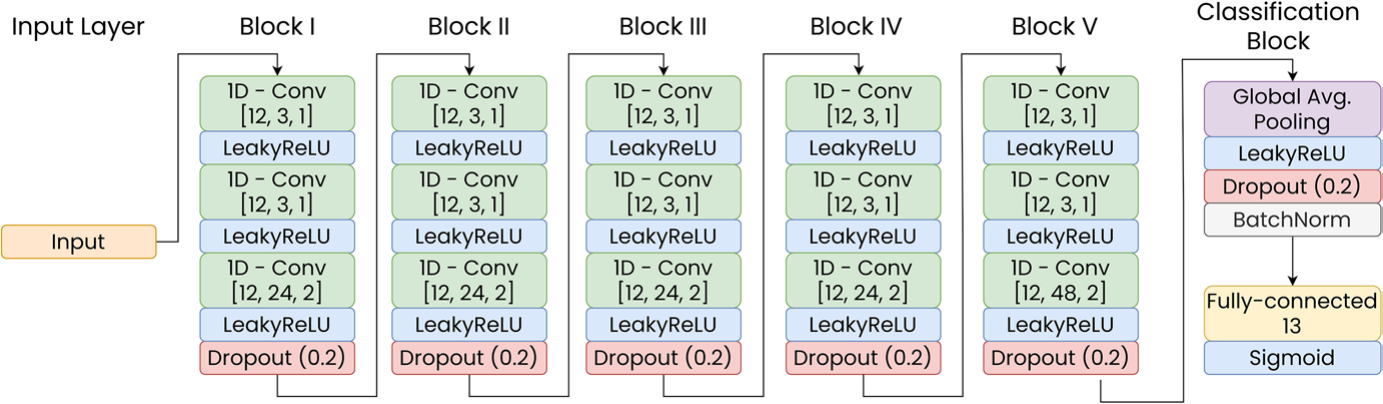
Model architecture: The input layer is followed by five convolutional blocks. Each block consisted of three 1D-convolutional layers with LeakyReLU activation (*α* = 0.3) and a concluding dropout layer. Square brackets indicate convolutional parameters: (filters, kernel size, stride). The final block was a global average pooling layer followed by LeakyReLU activation (*α* = 0.3), dropout, and batch normalization layer. The final block was concluded with a fully-connected layer with 13 units with sigmoid activation, serving as the classification layer

For this multi-class, multi-label classification task, a deep convolutional neural network with five one-dimensional convolutional blocks and a global average pooling prior to the classification layer was applied [46]. Figure 1 graphically summarizes the model architecture:

The model was trained with the binary cross-entropy loss function and the Adam optimizer [47]. The number of training epochs and learning rate decay, as suggested by Kingma et al. [47], are described in the individual methods’ descriptions. All implementations were executed in Python 3.7.4, and modelling was done with Tensorflow 2.4 [48].

### Learning Schemes

Eleven different learning schemes were applied (1 centralized baseline, 4 node-individual, 6 decentral), which are summarized in Table 3. The following chapters describe each method in detail.

**Table 3:** List of all applied learning schemes: 1 baseline model trained with all data centralized as performance reference (B), 4 individual models trained only with one of the four nodes’ datasets (I1–I4), and 6 decentralized learning schemes (M1–M3). The outcome description gives information about what each learning scheme results in. The references note the schemes origin or refer to similar algorithms found in literature

Notation	Name	Outcome description	Reference
B	Baseline model	1 model trained with centralized data of all nodes	*conventional ML*
I1-I4	I1: Individual model: CPSC	1 individual model trained with CPSC data only	*conventional ML*
	I2: Individual model: Georgia	1 individual model trained with Georgia data only	*conventional ML*
	I3: Individual model: INCART	1 individual model trained with INCART data only	*conventional ML*
	I4: Individual model: PTB	1 individual model trained with PTB data only	*conventional ML*
M1a	Regression ensemble	Classification obtained by averaging the results of I1-I4	[49]
M1b	Weighted regression ensemble	Classification obtained by averaging the results of I1-I4 with weights	[49]
M2a	Node-wise sequential learning	1 model trained on full data of nodes in one sequence	[42]
M2b	Batch-wise sequential learning	1 model trained on mini-batches of data in multiple sequences	*new*
M3a	Federated learning	1 model trained with standard federated learning (all nodes contribute equally)	[39]
M3b	Weighted federated learning	1 model trained with weighted federated learning (according to performance)	*new*

#### B: Baseline Centralized Model

The baseline model (B) served as a control classifier. For this model, all of the training data was joined to resemble a non-distributed optimal learning setting. On this aggregated training data, the model was trained for 50 epochs. Learning rate was decayed by Eq. 1 where the initial learning rates *lr*_0_ = 0.001, decay *λ* = 0.2, and current epoch number is *t*.

1 $$l{r}_t=\frac{l{r}_{t-1}}{1+t\ast \lambda }$$

#### I1-I4: Individual Models

One individual model was trained for each of the four data nodes, resulting in four additional models (I1: CPSC, I2: Georgia, I3: PTB, and I4: INCART), which were trained the same way as the combined model, but only with training data from the respective nodes.

#### M1a: Regression Ensemble

The first method to aggregate knowledge from federated data sources was to calculate the average of all classification results from the individual models I1–I4. All models trained on individual nodes were queried to classify the common test set. Subsequently, the result was determined by calculating the mean of each class-specific regression result. Finally, a threshold of 0.5 (= 50% probability) was applied to derive the classification result of M1a from the regression values.

#### M1b: Weighted Regression Ensemble

In M1a, all four individual models contributed equally to the final classification. However, in M1b, the individual regression results were weighted according to two factors (see Eq. 2): (a) their training set size proportion in relation the total dataset size (sample size *n*_*m*_ divided by the sum of sample sizes of all nodes) and (b) their node-internal AUROC performance.

2 $${w}_m=\frac{n_m}{\sum_{i=1}^4{n}_i}\ast \textrm{AURO}{C}_m$$

AUROC scores were interpolated to a range of [0, 1] and the final weights were normalized, so that the sum of all four weights was equal to 1.

#### M2a: Node-Wise Sequential Learning

**Fig. 2:**
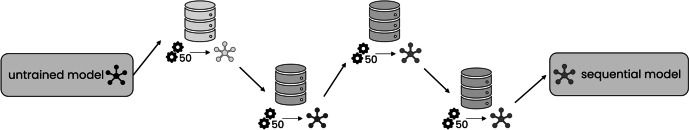
Node-wise sequential learning scheme (M2a): An untrained model was sequentially sent to all nodes, where it was trained for 50 epochs each. Learning rate was reset to 0.001 after each node

A combined model was trained by progressively exposing the initially untrained model to the data of one node after the other, so that knowledge was gathered sequentially. This method was comparable to Institutional Incremental Learning as proposed by Sheller et al [42]. For method M2a, a single model was sent to all nodes in the following order: (1) CSPC, (2) Georgia, (3) INCART, and (4) PTB, as depicted in Fig. 2.

At first, a model was initialized and sent to the first node, where the model is trained with the data of this specific node. After training, the model was sent to the next node in order, where its already partly optimized weights were the initial condition for continuing the training with the next pool of data. At each node, the model was trained for 50 epochs and the learning rate was decayed after each epoch as described in Eq. 1. After training at a node, the learning rate was reset to the initial value of 0.001 and sent to the next node in order. This was repeated until the model was trained at all nodes once.

#### M2b: Batch-Wise Sequential Learning

**Fig. 3:**
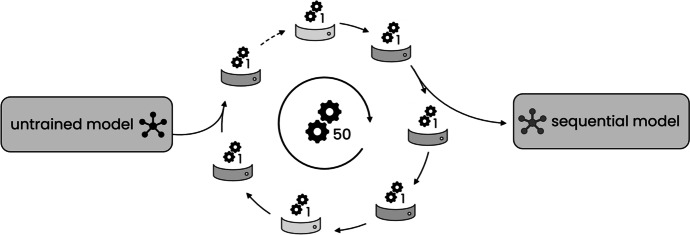
Batch-wise sequential learning scheme (M2b): An untrained model was trained on mini-batches at the nodes and passed on to the next node until all mini-batches were used (1 epoch). This was repeated for 50 epochs in total

To take the idea of sequential learning even further, we applied a novel method called Batch-wise sequential learning (M2b). Instead of fully completing training at a node like in M2a, the model was trained only on a randomly selected mini-batch of one node’s training data before sending it to the next node. The batch size of these mini-batches was set to 2% of a node’s training set size. This meant that each sample contributed equally to the model in M2b (which is equivalent to larger nodes consisting of more samples contributing more, as achieved with the weighted approaches). One epoch was considered completed when the model was exposed to each training sample exactly once. The model was trained for 50 epochs in total and the learning rate was decayed after each epoch according to Eq. 1. Method M2b is illustrated in Fig. 3.

#### M3a: Federated Learning

**Fig. 4:**
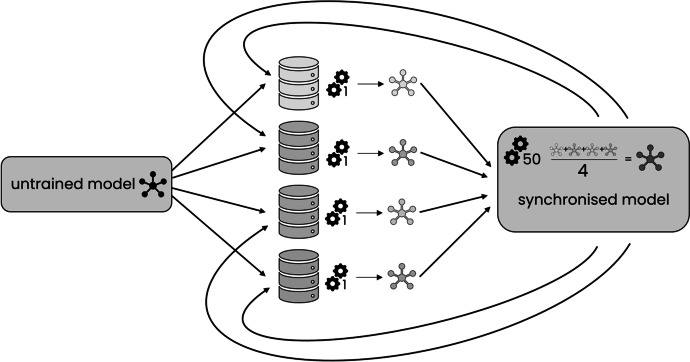
Federated learning scheme (M3a): In a first step, an initial, untrained model was distributed to all nodes, where they trained for 1 epoch, after which the models’ parameters were averaged. Subsequently, this average model was re-distributed to all nodes again starting a new update cycle. 50 of these update cycles were executed

In M3a, a model was trained in update cycles as depicted in Fig. 4. Each of these cycles repeated the steps as described in chapter 1: (1) distribute central model, (2) train locally at the nodes, (3) average the weights of the trained models, and (4) update central models with new parameters. The newly updated model from step 4 was then re-distributed as the central model in step 1 for the next update cycle. This method follows the original proposal for federated learning [39].

50 update cycles were completed. The epoch number for one cycle was set to 1. The learning rate was decayed according to Eq. 1, where *t* is the current update cycle iteration.

#### M3b: Weighted Federated Learning

As an advancement to federated learning (M3a), weighted federated learning (M3b) was implemented. A weighted average was used to calculate the new parameters in step 3 according to node-internal performance and dataset size as described in Eq. 2 for model M1b.

### Cross Validation and Evaluation Metrics

We trained models with a central dataset, with local datasets, and in decentral schemes. The only data available for training in the respective scheme was provided to the respective models during training. To find out how well all these models perform, each model was applied to a “global” test dataset, containing data from all nodes in a 10 fold cross-validation scheme.

During training in each fold N, 90% of each dataset was applied to the respective learning scheme. Depending on the learning scheme, training was carried out based on data from single nodes or from all nodes as described in the learning schemes chapter.

While training of fold N was done with different datasets depending on the learning scheme, all resulting models in fold N were evaluated with one and the same test-set-N. Therefore, the respective 10% shares of data from each dataset were aggregated to form one common test dataset N per fold. All models and decentral schemes described in the following chapters were tested on this test-set-N within fold N.

Predicted classes were compared to the known reference classes for each ECG, and each model was evaluated with six standard metrics for a complete assessment of classification performance: accuracy, area under the receiver operator curve (AUROC), Jaccard score, F1 score, specificity, and sensitivity. To correctly address the multi-label classification problem, the metrics (except accuracy) were derived from a weighted average according to the frequency of occurrence in the test set [50].

To combine the results achieved with each of these evaluation metrics in a representative way, we ranked the models by each of the six-evaluation metrics and calculated the mean ranks of all metrics for each model, i.e., the best model ended up with the lowest mean rank.

## Results

### Model Performance

Table 4 summarizes average values obtained during the 10-fold cross-validation process as achieved for the six-evaluation metrics (accuracy, AUROC, Jaccard score, F1 score, specificity, and sensitivity). Every model was ranked within each metric, and the average rank for each model was calculated.

**Table 4:** Detailed performances of all models and methods. Displayed numbers are mean values of metrics achieved during the 10-fold cross-validation scheme. Numbers in brackets note the achieved rank of performance and right column displays the average achieved rank

Model	Accuracy		AUROC		Jaccard Score		F1 Score		Specificity		Sensitivity		Average rank
B	0.915	**(1)**	0.872	**(1)**	0.392	**(1)**	0.532	**(1)**	0.731	**(1)**	0.517	(2)	**1.17**
I1	0.880	(10)	0.754	(10)	0.204	(10)	0.296	(10)	0.328	(10)	0.262	(10)	10
I2	0.888	(9)	0.769	(9)	0.273	(9)	0.345	(9)	0.415	(9)	0.317	(8)	8.83
I3	0.661	(11)	0.500	(11)	0.010	(11)	0.081	(11)	0.001	(11)	0.247	(11)	11
I4	0.890	(8)	0.817	(7)	0.336	(4)	0.487	(2)	0.529	(8)	0.551	**(1)**	5
M1a	0.903	(4)	0.835	(4)	0.302	(7)	0.376	(8)	0.646	(5)	0.313	(9)	6.17
M1b	0.903	(3)	0.837	(2)	0.344	(3)	0.441	(4)	0.558	(7)	0.456	(4)	4
M2a	0.893	(7)	0.802	(8)	0.300	(8)	0.415	(6)	0.650	(4)	0.381	(7)	6.67
M2b	0.906	(2)	0.836	(3)	0.350	(2)	0.472	(3)	0.724	(2)	0.449	(5)	2.67
M3a	0.900	(5)	0.818	(6)	0.327	(6)	0.391	(7)	0.568	(6)	0.390	(6)	6
M3b	0.895	(6)	0.823	(5)	0.328	(5)	0.441	(5)	0.703	(3)	0.490	(3)	4.5

Bolded values indicate the best score for each metric

### Average Rank per Model

**Fig. 5:**
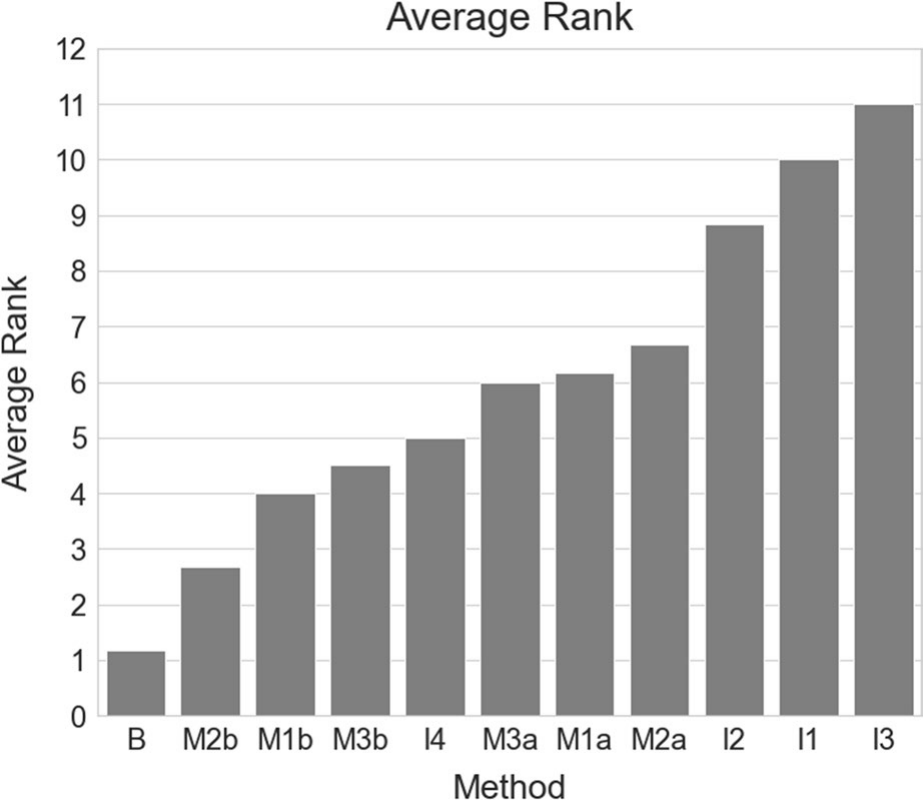
Average achieved rank: achieved rank of each model in sorted order

Figure 5 illustrates the average rank per model. As expected, the baseline model with all data pooled centrally during learning performed best. From all decentral learning methods, models taking the size of the different nodes into account performed best (M1b, M2b, M3b), while models derived on data from small nodes only performed worst (especially I3).

### Evaluation Metrics per Model

**Fig. 6:**
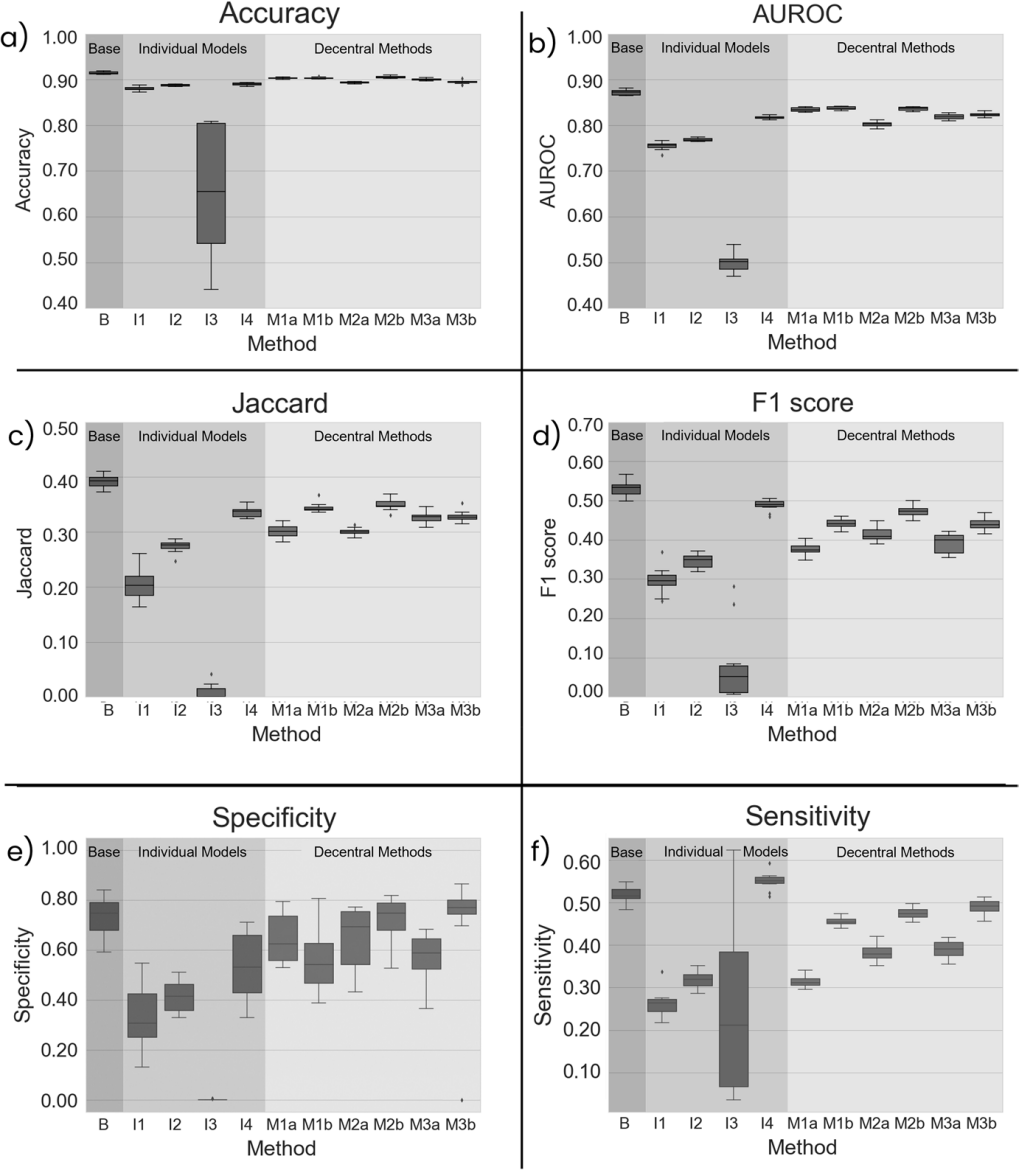
Model performances of all tested learning schemes: Figures **a**–**f** are box-whisker-plots of all recorded evaluation metrics: accuracy (**a**), AUROC (**b**), Jaccard score (**c**), F1 score (**d**), specificity (**e**), and sensitivity (**f**). Error bars indicate minimum and maximum values. Small rhombus symbols denote outliers

The evaluation metrics are displayed as box-whisker-plots for graphical comparison in Fig. 6.

## Discussion

Privacy-preserving artificial intelligence (PPAI) is widely discussed nowadays. Federated learning is commonly used for training models based on data from large user groups (e.g., mobile phone users). The goal is to aggregate knowledge without centralizing data to protect personal information. Clinical scenarios are different from the usual federated learning approach (e.g., less data nodes, less data at the nodes, non-iid data), and thus, implementing this principle requires adaptions. We have implemented six different decentralized learning schemes for ECG classification and compared the results of these learning schemes with each other and with a centralized approach.

Figure 5 gives a summarized overview over all applied learning schemes. As expected, the baseline model (B), which was trained on all available data performed the best and thus its performance serves well as reference. All individual models (I1–I4) performed worse than the baseline model. The INCART dataset (I3) was arguably too small (*n* = 74) to produce any sensible deep classification model on its own. I4 performed the best of all individual models. This can be explained by the fact that I4 was trained on the largest dataset (PTB-XL and PTB, 51.86% of the entire dataset), and therefore, the test dataset also consisted of more than 50 % from this specific dataset, constituting a bias in the test set towards the I4 model. Of all decentralized learning methods, the novel batch-wise sequential learning (M2b) and weighted regression ensemble (M1b) performed best (see Fig. 5). Although M1b achieved a higher AUROC score, M2b was overall the better performing model when considering all metrics. M2b was designed to mimic standard machine learning as close as possible in a decentral scenario, and thus, it appears sensible that it performed best out of the tested decentralized algorithms.

Furthermore, our weighted variant of the federated learning scheme (M3b) outperformed the standard unweighted algorithm (M3a). The benefit of weighting is assumed to be related to the imbalance of the node size and the heterogeneity of the different datasets (i.e., cancelling out the negative impact of bad performing models trained on small datasets). Since in our experiments, one of the nodes was significantly smaller than the others, and since the number of events per class varied a lot among the nodes, weighting led to significantly better results. In a more homogeneous and balanced setting, however, the effect is expected to be less severe.

The novel methods of batch-wise sequential learning (M2b) and the weighted federated learning (M3b) both outperformed standard federated learning, although the latter was exceeded by the weighted regression ensemble (M1b).

As shown in Fig. 6, performance varies across the statistical measures. Accuracy proved to be a suboptimal measure of performance due to the sparsity of labels. Most entries in the label vectors were negative (i.e., a diagnosis was not present), and thus, a model predicting only negatives would achieve high accuracy scores. To address this inadequacy, the Jaccard score was used which gauges in how many samples all classes were predicted correctly. This scenario with 13 classes and multi-label possibilities is a highly complex classification problem, and thus, no model achieved a Jaccard score above 0.4. The sparse label problem can also be seen in specificity and sensitivity scores. Due to overhang of negative samples, models were incentivized to be conservative with classification (i.e., preferring negative predictions over positives). This naturally led to a low false positive rate, which resulted in higher specificity than sensitivity. Assessing individual model performances, the unreliability of I3 is visible in all metrics. Furthermore, a trend becomes apparent that the variant schemes (M1b, M2b, M3b) performed better than their more conventional counterparts (M1a, M2a, M3a). The variants take dataset sizes and internal performance into account which appears to a valuable consideration. To add to that, the variants also tend to be more stable and have less variance in their performance across the 10 cross-validation folds.

### Technical Implications

As compared to centralized approaches, all decentral schemes require computational power at the nodes, which comes with some challenges for the respective healthcare providers: firstly, the nodes need to be online and ready for training simultaneously (especially true for M2b, M3a, and M3b) and secondly, models are exchanged at a high frequency (most notably M2b), which might cause significant network loads at the nodes. In a real application, this could be addressed by nightly routines, where network and computational loads are typically lower. However, comparing the decentralized methods’ computational costs with those of the baseline model is most interesting and most relevant for real applications.

The regression ensemble methods (M1a and M1b) cause low network load and have almost no additional computational cost compared to conventional machine learning. They could potentially be more efficient since model training can be parallelized. The only additional operation required is the consolidation of all individual predictions, which is computationally inexpensive. The federated learning schemes (M3a and M3b) also parallelize model training, but the frequent update cycles slow down the optimization process and cause substantial network load. The degree of this effect depends on a multitude of factors (e.g., resources at the nodes, network availability, network speed) and is difficult to assess, but the overall computational cost is likely to be higher than in conventional machine learning. M3b is slightly more costly than M3a due to the extra step of finding weights and weighing the average accordingly. The sequential learning schemes (M2a and M2b) serialize training instead of parallelizing it. The computational cost is expected to be approximately the same as conventional learning, but is slowed down by exchanges over the network. Node-wise sequential learning (M2a) is less demanding on the network than the batch-wise variant (M2b), which constitutes the highest network load of all tested schemes. In our implementation, a simple method of calculating weights was used, which ultimately has minimal impact on the overall optimization duration. However, more complex weight calculations could constitute a more substantial portion of model training and should be carefully considered.

### Limitations

Due to its size, the INCART dataset proved impractical for machine learning purposes and models trained solely on this dataset did not generalize well (see Table 4 and Fig. 6) as performance on the test data was poor. However, it was included mainly to investigate the question whether institutions with small amounts of data could have a negative impact on a decentralized learning scheme. Using the dataset size as a factor to weigh the influence of individual models according to their dataset size as in Eq. 1 improved performance as the weighted variants of M1 and M3 performed better than their unweighted counterparts. This result might indicate that participants with inadequately small datasets can potentially cause more harm than good in a decentralized learning scheme. However, it remains unclear, whether the size alone is the cause of this effect as the INCART set was also different in ECG length and average patient age. Furthermore, the increase in performance could not only stem from giving the INCART set less influence but also from giving the PTB dataset more importance.

**Fig. 7:**
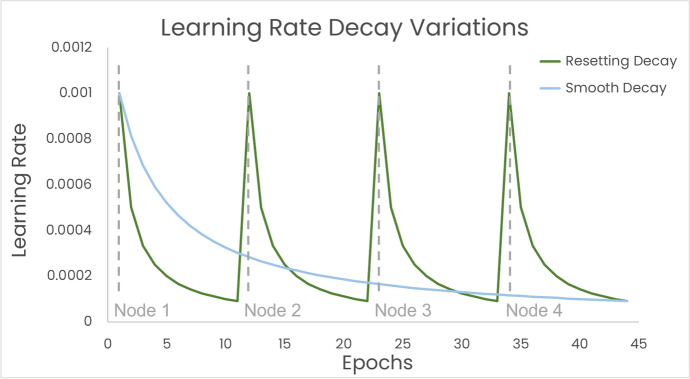
Schematic explanation of potential learning rate decay options: the green line represents the learning rate behavior as executed in M2a. The blue line shows a variant where the learning rate is decaying smoothly

For schemes M2a and M2b, the order of nodes may have a detrimental influence on the final results, potentially leading to two possible extremes: (A) the first node applied on the model might have already optimized the model’s parameters towards a local optimum in such a way that other nodes cannot re-adjust them to the global optimum anymore. (B) The influence of the first node might be completely extinguished by the other nodes at the end and cause its gradients’ change to be irrelevant. This effect is expected to be more prominent in M2a as compared to M2b, as each node is only used once in M2a. The likelihood of extreme A or extreme B to occur can be adjusted by the learning rate decay. In this experiment, we have reset the learning rate whenever the model was passed over to a new node, making M2a more prone to extreme B. Not resetting the learning rate and decaying it smoothly from node to node might lead to better overall results, although this variant would be more susceptible to extreme A. This matter still needs to be explored in the future. Both versions of learning rate decay are explained schematically in Fig. 7.

For a real-world implementation, the weighted regression ensemble scheme seems highly attractive since it performed well and is computationally inexpensive. However, all the models were currently trained with the same settings (model architecture, number of epochs, learning rate, batch size, etc.) and method of calculating the weights per node (based on size and intra-node AUROC). After the training, the averaging weights represent the only meta-parameter that can still be adapted. Because only the models’ regression outputs are used for M1b, individualizing the models and training routine for each specific node could ultimately lead to better results. Therefore, more experiments with different settings for the individual datasets are possible subjects for further research.

Besides technical limitations, the properties of the used data must be considered. By nature, ECG signals are quasi-periodic, which might impact the classification algorithms’ performance. How well the insights found in this study translate to non-periodic signals remain unexplored and could be subject of future analysis. Furthermore, for the present work, no specific pre-processing steps were taken into account for the periodically occurring QRS complexes, which might have improved overall classification results. Li and Clifford showed that constructing individual-specific templates of periodical patterns in physiological data (photoplethysmography) with dynamic time warping can be helpful in classification problems [51].

### Outlook

Future studies might focus on the influence of hyper-parameters on optimization. Additionally, different application scenarios with regression, single label classification, more nodes, more and/or less imbalanced, and/or heterogeneous nodes could further elucidate the advantages and disadvantages of all learning schemes.

To investigate the underlying causes for the performance increases in the weighted variants of M1 and M3, analyses stratifying dataset sizes or simply excluding the INCART dataset entirely could provide more insight into the exact mechanisms of performance reduction with small or otherwise heterogeneous datasets.

Furthermore, the applied classification algorithms used in this study could be used with non-periodic signals like electroencephalography (EEG) or electromyography (EMG) data. Addressing EEG and EMNG in a follow-up study could provide further insight into the algorithms’ performance regarding stationarity because EEG signals are non-stationary, while EMG signals are typically stationarity. While the model type (convolutional neural networks) is likely to be suitable, minor adjustments in model architecture might be required to ensure satisfactory performance.

## Conclusion

Depending on the application scenario, different learning schemes are suitable. While a central approach should be preferred whenever legally and ethically possible, decentral schemes carry considerable potential in scenarios where privacy is of utmost priority. We have shown that the principle of federated learning as a form of privacy-preserving AI is indeed applicable to decentral ECG data. Since federated learning was designed for the application with millions of nodes (e.g., smartphones), its application to healthcare data might require adaptions due to the different characteristics (e.g., less nodes, more heterogeneous). We have demonstrated such adaptions and variations of standard federated learning can improve performance. The properties of each data node should especially be taken into consideration by the decentral algorithms (e.g., weighting the impact of each individual node). This increased performance most, which is necessary to develop successful decentral learning applications, which could constitute a valuable step towards privacy-preserving applications of AI in healthcare data.

## Data Availability

All data used in this study is open access. Links to the six datasets can be found here:

**Table:** 

CPSC	CPSC-Extra	INCART	PTB	PTB-XL	Georgia Database (from: https://physionetchallenges.org/2020/)

## Code Availability

All programming code (Python) will be made available upon request. Additionally, all code will be released as open-source on GitHub in case of successful publication.
